# Diagnostic dilemma in postpartum associated hemolytic uremic syndrome in a 38th week pregnant 31-year-old Congolese: a case report

**DOI:** 10.1186/s12884-020-03185-3

**Published:** 2020-08-27

**Authors:** Marc Tshilanda, Ulrick Sidney Kanmounye, Céline Tendobi, Freddy Mbuyi

**Affiliations:** 1 Department of Obstetrics and Gynecology, Centre Hospitalier Mère-Enfant (CHME) Monkolé, Kinshasa, Democratic Republic of Congo; 2 Faculty of Medicine, Université Notre-Dame du Kasayi, Kananga, Democratic Republic of Congo; 3 Faculty of Medicine, Université Technologique Bel Campus, Kinshasa, Democratic Republic of Congo; 4 Faculty of Medicine, Université de Kinshasa, Kinshasa, Democratic Republic of Congo

**Keywords:** Atypical hemolytic uremic syndrome, Differential diagnosis, Microangiopathic hemolytic anemia, Postpartum complications, Thrombotic microangiopathy

## Abstract

**Background:**

Thrombotic microangiopathy is associated with HELLP syndrome, thrombotic thrombocytopenic purpura, or atypical hemolytic uremic syndrome (aHUS) during pregnancy. Standard laboratory and physical examinations can help distinguish between these three diseases promptly and guide their management. This is critical because their managements and prognoses differ considerably. The ADAMTS13 test, complement tests, and biopsies can help ascertain the diagnosis; however, they take time, and are not widely available. In this case report, we present a case that highlights the diagnostic and therapeutic dilemmas associated with the aforementioned diseases.

**Case presentation:**

A 31-year old P3G3 patient presented at 38 weeks with high blood pressure, bilateral pitting edema, and a low fetal heart rate. A cesarean section was performed to extract the fetus. On postoperative day 2, the suites were marked by anemia, low platelet count, acute kidney injury, declining liver function, and the presence of schistocytes on the peripheral thin smear. The patient was lucid, coherent, and presented no neurological deficits. The ADAMTS13 test and anti-complement therapy were not readily available, so the team made a presumptive diagnosis of aHUS based on the history, clinical presentation, and standard laboratory results. Due to a lack of anticomplement therapy, the patient was prescribed four sessions of hemodialysis. The renal function and platelet count gradually increased, and the patient was discharged on postoperative day 18. The patient was followed for over a year and did not present relapses of thrombocytopenia or microangiopathic hemolytic anemia.

**Conclusions:**

The prompt diagnosis and management of aHUS lead to favorable outcomes. Healthcare providers should be able to rapidly differentiate between pregnancy-associated thrombotic microangiopathies and prescribe appropriate management. Here, we highlighted the challenges of diagnosing and managing postpartum associated aHUS in a low-resource setting.

## Background

Thrombotic microangiopathy (TMA) during pregnancy and the postpartum is most often associated with HELLP syndrome (hemolysis, increased liver enzymes, low platelets) or preeclampsia with severe manifestations [1–3]. Rarely, TMA may be due to thrombotic thrombocytopenic purpura (TTP), complement-mediated TMA, or atypical hemolytic uremic syndrome (aHUS). Pregnancy associated microangiopathic disorders all have distinct definitions and prognoses (Table 1). However, they are often misdiagnosed and managed inadequately.

**Table 1:** Comparison of pregnancy-related microangiopathic disorders - atypical Hemolytic Uremic Syndrome, Hemolysis Elevated Liver enzymes, Low Platelet count, preeclampsia, and thrombotic thrombocytopenic purpura. The frequency of presentation of the characteristics are indicated in ascending order: uncommon, seldom, sometimes, frequently, and always

Characteristic	aHUS	HELLP syndrome	Preeclampsia	TTP
Onset	Postpartum	Third trimester	Third trimester	Throughout pregnancy and postpartum
MAHA	Frequently	Frequently	Seldom	Always
Thrombocytopenia	Frequently	Frequently	Seldom	Always
Hypertension	Uncommon	Seldom	Always	Uncommon
DIC	Seldom	Always	Seldom	Uncommon
Liver disease	Seldom	Always	Seldom	Seldom
Renal disease	Always	Sometimes	Sometimes	Seldom
CNS disease	Seldom	Seldom	Seldom	Always
Management	Anticomplement therapy	Delivery and transfusion of blood products	Delivery	Plasmapheresis

*aHUS* atypical Hemolytic Uremic Syndrome, *CNS* Central Nervous System, *DIC* Disseminated Intravascular Coagulation, *HELLP* Hemolysis, Elevated Liver enzymes, Low Platelet count, *MAHA* Microangiopathic Hemolytic Anemia, *TTP* Thrombotic Thrombocytopenic Purpura Com

The complexity of managing TMA cases during pregnancy is increased by a lack of expertise, tests, or equipment needed to diagnose and treat these conditions promptly. Moreover, most patients from developing countries pay for their healthcare out-of-pocket. Resultantly, clinicians in low resource settings factor non-clinical aspects when they decide on the management of their patients. This case study aims to draw attention to the difficulties associated with the diagnosis and management of pregnancy-related atypical hemolytic uremic syndrome in low-resource settings.

## Case presentation

A 31-year-old black Congolese female patient, P3G3, who had a pregnancy of 38 weeks and four days, was transferred from a district hospital to a tertiary facility in Kinshasa, Democratic Republic of Congo, for severe preeclampsia with acute on chronic fetal distress. Her past medical history was notable for eclampsia during her first pregnancy in 2011 and preeclampsia in 2013 during her second pregnancy. She had had two cesarean sections for her pregnancies, and she had her antenatal care for her third pregnancy at a referral hospital. She was diagnosed with preeclampsia during her third pregnancy for which she received 250 mg alpha-methyl-dopa twice a day. ​​During the 30th week of gestation, the patient’s systolic blood pressure became labile, oscillating between 140 and 150 mmHg, despite her antihypertension medication. The patient consulted at a district hospital where she underwent a fetal wellbeing ultrasound which did not find anomalies.

The patient was the 7th of 9 children, and her father was hypertensive. She weighed 72 kg for 155 cm, and upon arrival at the authors’ hospital, her blood pressure was 217/152 mmHg. Her heart rate was 101 bpm, her respiratory rate was 24 cpm, and SpO2 was 96% free air. The patient was in pain, she was lucid and coherent, her palpebral conjunctivae were colored, and she had bilateral pitting edema. The fundus height was at 30 cm, the presentation was cephalic, the fetus was bradycardic at 88 bpm, and there were no signs of genital bleeding. The cervix was median, soft, 80% effaced with a 2 cm dilation. Urine deep stick revealed 3+ proteinuria. She had 1.5 mg/dL of creatinine (normal: 0.5–1.5 mg/dL), 22 mg/dL of urea (normal: 10–50 mg/dL), 15,000 white blood cells/ml, 213,000 platelets/ml and 14 g/dL hemoglobin. Based on these findings, we indicated an emergency cesarean section for acute fetal distress, which resulted in the extraction of a dead infant.

Postoperative suites were marked on day two by decompensated anemia (hemoglobin at 7.8 mg/dL) for which the patient was transfused two units of packed red blood cells. On postoperative day three, the patient presented an abdominal effusion, exacerbation of the bilateral pitting edema, blood pressure increase, hematemesis, melena, petechiae, hematuria, and oliguria. Her blood pressure was 215/120 mmHg and she had signs of renal failure (creatinine = 6.9 mg/dL (normal: 0.84–1.21 mg/dL); urea 132.5 mg/dL (normal: 5–20 mg/dL); hyperkalemia at 6.4 mmol/L (normal: 3.5–5 mmol/L); hyponatremia 109 mmol/L (normal: 136–145 mmol/L); hypocalcemia 0.88 mmol/L (normal: 1.12–1.32 mmol/L)), and signs of hepatic failure (AST 135 IU/L, normal: < 33 IU/L; ALT 325 IU/L, normal: < 33 IU/L; prothrombin ratio 100% (normal: 80–110%). PTT was 39 s (normal: 24–35 s), LDH was 1398 IU/L (normal: 120–280 IU/L), and total bilirubin was 0.35 mg/dL (normal: 0-1 mg/dL)). She equally had neutrophilic leukocytosis at 22,180 cells/mm^3^ and low platelets at 44,000 cells/mm^3^. Additionally, schistocytes were identified in the peripheral thin smear.

**Fig. 1:**
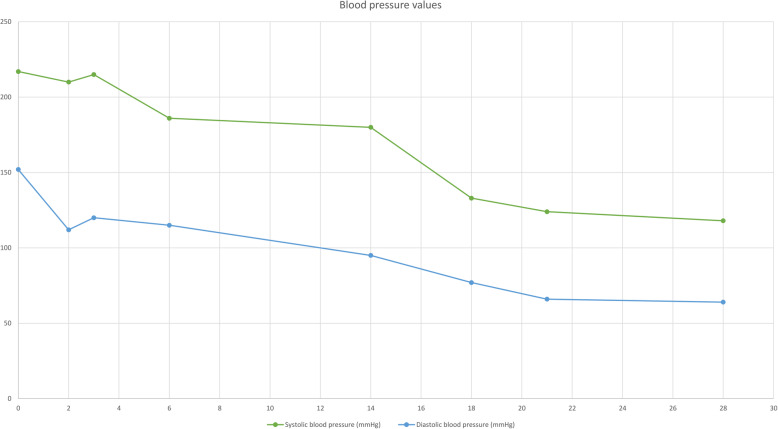
Blood pressure values from postoperative day 1 to day 28. The green line illustrates the evolution of the systolic blood pressure, and the blue line represents the evolution of the diastolic blood pressure

**Fig. 2:**
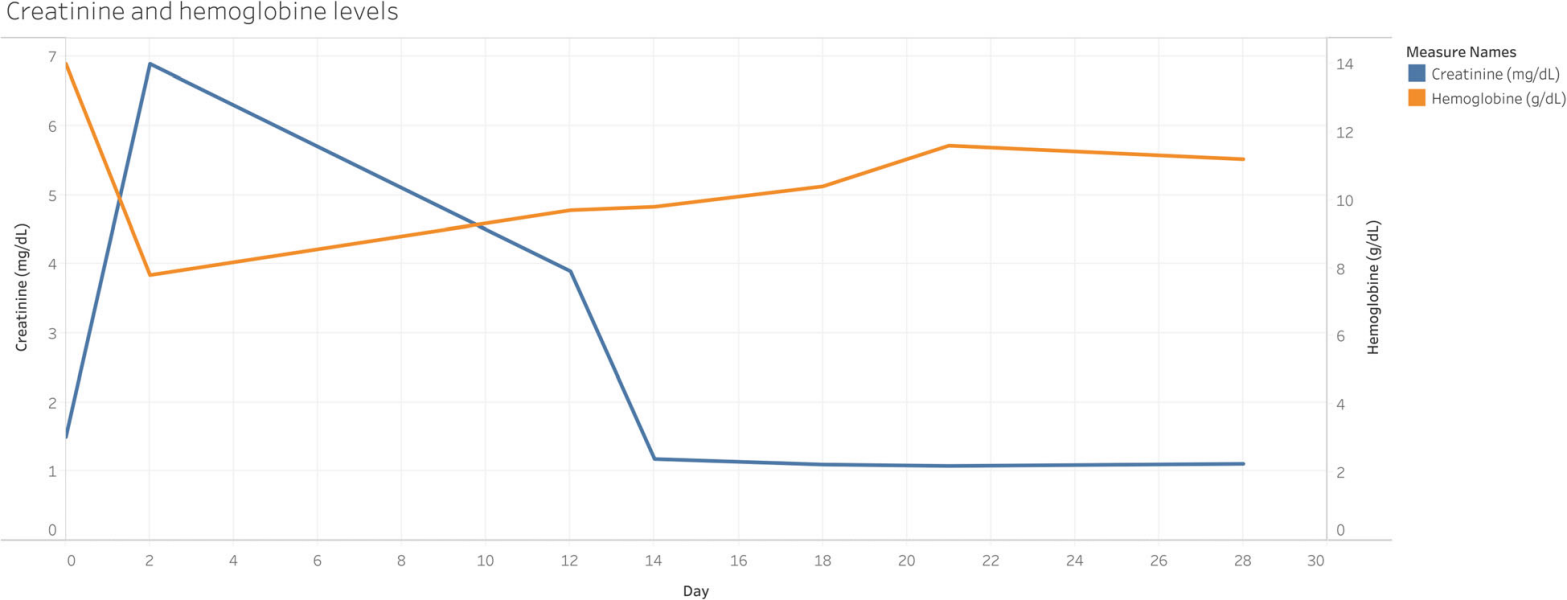
Serum creatinine and hemoglobin values from postoperative day 1 to day 28. The left axis and the blue line represent the creatinine values. The right axis and the orange line represent the hemoglobin values

**Fig. 3:**
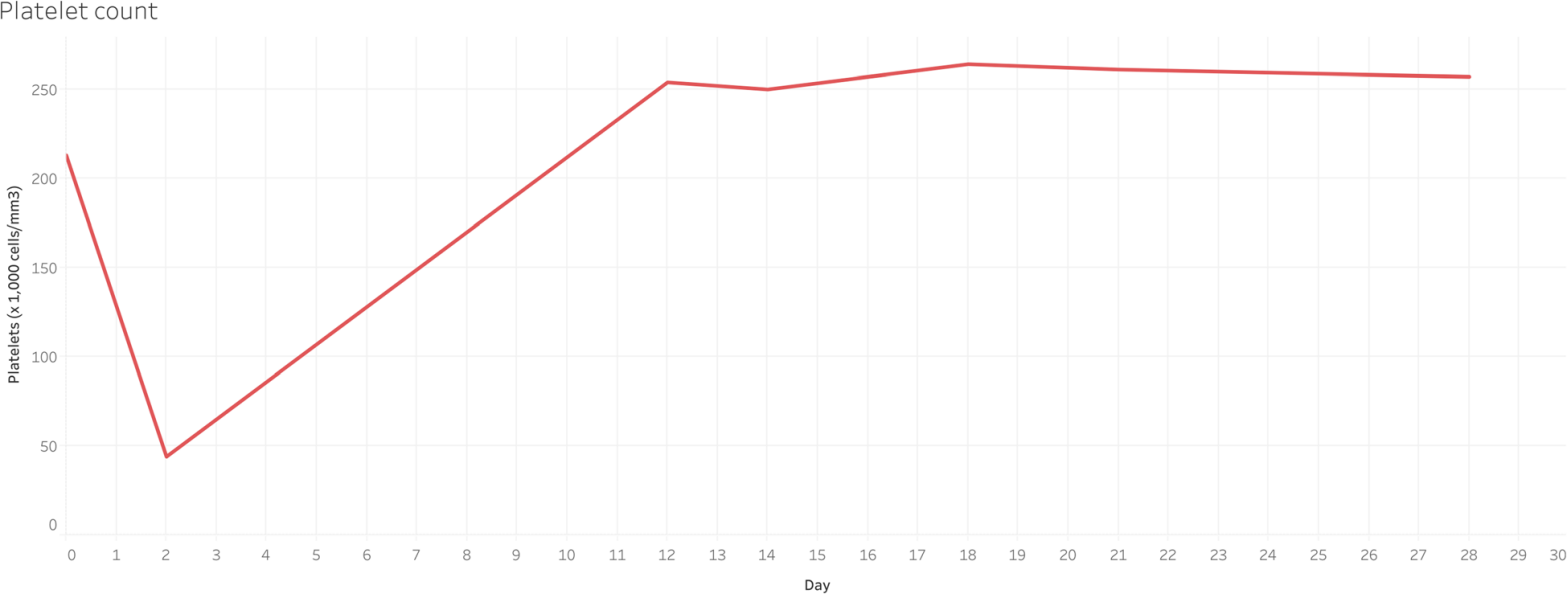
Platelet count from postoperative day 1 to day 28. The red line represents the evolutions of the platelet count

The team excluded TTP and HELLP syndrome as possible causes of the postpartum microangiopathic hemolytic anemia (MAHA). This decision was based on the history, clinical presentation, and laboratory findings. Atypical hemolytic uremic syndrome was retained as the final diagnosis, and in the absence of anti-complement therapy, the patient underwent four sessions of hemodialysis. Hypertension was treated with Nicardipine, 5 mg/hr. IV with a 2.5 mg/hr. increase every 15 min without exceeding 15 mg/hr., and the goal was to lower the systolic blood pressure below 160 mmHg. Hyperkalemia was corrected with insulin and glucose (10 units of insulin dose with 25 g of glucose per each 1 mmol/L of potassium above the normal). A favorable clinical and biologic evolution was observed, and the patient was released for outpatient follow-up on postoperative day 18 (Figs. 1, 2 and 3).

Laboratory tests could not be obtained more frequently due to their financial burden to the patient and her immediate family. The patient had a normal kidney function at postoperative day 160 (urea 24.2 g/dL, creatinine 0.8 mg/dL) and postoperative day 202 (urea 16.9 g/dL, creatinine 0.8 mg/dL). She did not present new episodes of microangiopathic hemolytic anemia.

## Discussions and conclusions

In low resource settings, the diagnosis and treatment of patients with MAHA-associated diseases can be challenging. While some biological tests can help confirm the diagnosis (ADAMTS13, C3b, and factor H), these are often unavailable in low resource settings and can delay patient management [4]. For this reason, healthcare providers rely on the history and clinical presentation to distinguish between these diseases. For example, sudden onset of anemia, thrombocytopenia, and renal insufficiency are in favor of HELLP syndrome. Progressive worsening of the renal lesion is highly suggestive of aHUS while the presence of minimal renal insufficiency, severe thrombocytopenia with signs of neurological involvement pleads in favor of TTP [2].

Worsening clinical signs of gestational hypertension, preeclampsia, or the aggravation of anemia, thrombocytopenia, and renal function abnormalities (most common target organ damage) after delivery point towards TTP and aHUS. While minimal renal impairment with severe thrombocytopenia and neurological signs suggest TTP and the need for plasma exchange therapy, progressive renal injury (in the absence of an identifiable cause of acute tubular necrosis) suggests aHUS and the need for anti-complement treatment [1, 5].

Unfortunately, this approach is not infallible. For example, thrombocytopenia develops in 5 to 10% of women during pregnancy or the immediate postpartum. A low platelet count is often a fortuitous feature and can, in some instances, be misleading, such as in women with coexisting systemic or gestational disorders. As a result, low platelet count might lead to an indication of a maternal intervention that can harm the fetus [6].

Given the history and presentation, in this case, there are three possible scenarios we must consider: gestational hypertension or preeclampsia triggering the complications of aHUS; gestational hypertension or preeclampsia evolving to the HELLP syndrome; or aHUS initially presenting as hypertension during pregnancy progressing to overt complications. Gestational hypertension is “hypertension that appears de novo after 20 weeks’ gestation and normalizes after pregnancy”. In comparison, preeclampsia is defined as “de novo hypertension after 20 weeks’ gestation accompanied by at least one of the following: proteinuria, maternal organ dysfunction [renal, hepatic, neurological, and hematological] and uteroplacental dysfunction” [7]. The patient had a history of eclampsia during her first pregnancy, and she presented with bilateral pitting edema and proteinuria. Therefore, she had preeclampsia. At presentation, the patient did not have anemia or low platelets. However, more than 24 h after she had delivered her baby, she had hemorrhages, anemia, declining liver, and renal functions. Standard laboratory findings can help differentiate HELLP syndrome, which is a clinical presentation of severe preeclampsia from aHUS. Women with HELLP syndrome present with LDH (438–782 IU/L) and creatinine (0.7–1.1 mg/dL) levels that are significantly lower than their counterparts with aHUS (LDH 1325–3940 IU/L and creatinine 3.9–7.6 mg/dL). On the other hand, women with HELLP syndrome present higher hemoglobin (7.3–10.4) and platelets (47,000-82,000) [8].

Our patient had 1398 IU/L LDH, creatinine (6.9 mg/dL), hemoglobin (7.80 g/dL) and platelets (44.000 cells/mm^3^). Also, HELLP syndrome and preeclampsia tend to resolve once the patient delivers her baby. The elements mentioned above were all in favor of preeclampsia, triggering the complications of aHUS. However, the favorable evolution of the patient after just four hemodialysis sessions is not in favor of preeclampsia, triggering the complications of aHUS.

In the absence of confirmatory genetic test results or kidney biopsy to demonstrate the pathology of thrombotic microangiopathy, the diagnosis of aHUS remains presumptive. We recognize that the clinical and lab criteria are not ideal; however, in most hospitals in developing countries, these are readily available and can help inform clinicians as they make time-sensitive decisions.

aHUS presents as a triad of MAHA, thrombocytopenia, and acute renal failure and can lead to hypertension and extrarenal organ dysfunction [9, 10]. Kidney injury is the result of lesions to endothelial cells by the membrane attack complex, C3a, and C5a. When these lesions affect endothelial cells of preglomerular arterioles, they can lead to the dysfunction of the juxtaglomerular apparatus and subsequently to severe but unstable hypertension.

aHUS, unlike “typical” hemolytic uremic syndrome, is not caused by the Shiga toxin and is not preceded by hemorrhagic diarrhea. The prodomeless aHUS is due to a defect in the alternative complement pathway regulation [9]. The alternative complement pathway is regulated by proteins found in the plasma and on the surface of host cells. Mutations of regulatory proteins predispose pregnant women to develop aHUS and are responsible for the evolution of HELLP syndrome or preeclampsia to aHUS in pregnant women [10].

The C3a and C5a released in the kidney can equally leak into the general circulation and stimulate the release of histamine by basophils causing lesions of non-renal end organs such as the brain, the retina, the bronchus, the intestine, the pancreas, and serous membranes [9]. MAHA in aHUS results from mechanical injury of erythrocytes as they go through stenotic arterioles and capillaries. The degree of hemolysis depends on the severity of the stenosis in the arterioles and capillaries. Consequently, it is not uncommon for patients with aHUS to present without MAHA when the stenosis is minimal [9]. The last sign of the aHUS triad, thrombotic thrombocytopenia, results from the consumption of platelets.

It is difficult to ascertain the diagnosis of aHUS, and delays in diagnosis and treatment can be life-threatening [2, 9]. The results of molecular tests can take weeks to be available, and they might return false negatives. In practice, patients with TMA are treated with plasma exchange therapy until all non-aHUS causes of TMA have been excluded. As soon as all non-aHUS causes of TMA have been eliminated, aHUS is retained as the diagnosis, and the patient is switched to complement therapy [9]. The reason being that plasma therapy is ineffective in aHUS patients with mutations of complement system regulatory membrane proteins. Additionally, overall dialysis free survival at one year of aHUS patients treated with plasma therapy is only 40% [9]. Unlike plasma therapy, complement therapy is effective in all aHUS cases, indiscriminate of the membrane protein mutations, and failure of the complement therapy should prompt the reevaluation of the diagnosis [11]. Patients are put on eculizumab, a humanized monoclonal antibody of complement C5, once a week for five weeks, then fortnightly subsequently [9].

Fortunately, the evolution was favorable after hemodialysis in this case. Clinicians must be able to recognize the signs of TMA early on and to narrow down the diagnosis to aHUS. However, governments must invest more in the health system to provide underresourced physicians with the tools to conveniently manage diseases such as aHUS.

## Data Availability

The datasets used and/or analyzed during the current study are available from the corresponding author on reasonable request.

## Abbreviations

aHUS: atypical Hemolytic Uremic Syndrome
MAHA: Microangiopathic Hemolytic Anemia
TMA: Thrombotic Microangiopathy
TTP: Thrombotic Thrombocytopenic Purpura
